# Interpretable Event-Driven Multisensor Risk-Evolution Analysis for Methane Early Warning

**DOI:** 10.3390/s26134126

**Published:** 2026-06-30

**Authors:** Shuze Li, Yang Yang, Zhilei Wu, Rong Xiao

**Affiliations:** 1 School of Management, China University of Mining and Technology-Beijing, Beijing 100083, China; libham1999@foxmail.com (S.L.); bwu_yangyang@126.com (Y.Y.);; 2 Information Institute of the Ministry of Emergency Management of the PRC, Beijing 100029, China

**Keywords:** multisensor monitoring, methane early warning, risk evolution, temporal dependency analysis, event-driven analysis, industrial anomaly detection, interpretable monitoring, underground coal mines

## Abstract

Methane exceedance events in underground coal mines are often associated with progressive multisensor abnormal evolution processes involving operational, ventilation, environmental, and methane-drainage subsystems. Existing studies primarily focus on methane concentration prediction and provide limited interpretability regarding how abnormal evolution patterns emerge before threshold exceedance. To address this limitation, this study proposes an interpretable event-driven multisensor risk-evolution analysis framework for methane early warning. Methane exceedance events are first extracted from multisensor monitoring data, and a continuous multisensor risk representation together with a persistence-based trigger mechanism is developed to identify sustained abnormal evolution prior to methane exceedance. Event-specific temporal dependency networks are then constructed using lagged dependency analysis to characterize multisensor interaction structures within event windows. Representative evolution paths and recurrent critical variables are further identified to reveal interpretable abnormal evolution patterns. Experiments conducted on a real underground coal mine monitoring dataset containing 784 methane exceedance events demonstrate that the proposed framework achieved the highest early-warning performance among all compared baselines. Under the Any-Target-Sensor criterion, the framework attained an effective warning rate of 0.848 and significantly outperformed benchmark methods in event-level McNemar tests (*p* < 0.001). The results further indicate that methane exceedance events are generally associated with structured multisensor abnormal evolution processes rather than isolated methane fluctuations, providing an interpretable system-level perspective for methane early warning.

## 1. Introduction

Methane hazards remain one of the most critical safety concerns in underground coal mine operations. Due to confined underground environments, dynamic ventilation conditions, and continuously changing operational activities, methane accumulation may occur rapidly once gas emission exceeds local ventilation capacity. Methane exceedance events are often associated with coupled variations in ventilation states, environmental conditions, methane drainage processes, and operational activities, potentially leading to severe accidents and production disruptions [1,2]. Understanding how abnormal states evolve before methane threshold exceedance is therefore essential for improving safety monitoring and supporting proactive intervention in underground mining systems.

Modern underground monitoring systems continuously generate large-scale multisensor time-series data, including methane concentration, airflow velocity, environmental parameters, drainage conditions, and operational variables. The increasing availability of high-frequency industrial monitoring data has significantly promoted the development of data-driven monitoring and anomaly analysis methods in complex industrial systems [3,4]. Compared with traditional single-variable threshold alarms, multisensor monitoring provides opportunities for analyzing system-level abnormal evolution patterns and temporal interactions among heterogeneous variables.

Existing methane monitoring studies have mainly focused on concentration prediction and warning tasks. Early approaches primarily relied on statistical forecasting and empirical time-series analysis [5]. With the increasing availability of underground monitoring data, various machine-learning and deep-learning approaches have been introduced for methane concentration prediction and gas early warning in coal mines. Artificial neural networks, recurrent neural networks, long short-term memory networks, and hybrid optimization–learning frameworks have demonstrated promising performance in short-term methane forecasting under complex mining conditions [6,7,8]. Recent studies have further explored graph-neural-network-based prediction models and multisensor early-warning systems to capture spatiotemporal dependencies among underground monitoring variables [9]. Recent review studies have highlighted the rapid adoption of artificial intelligence techniques in methane prediction, gas outburst warning, and intelligent coal mine safety monitoring [10].

Beyond coal mine methane monitoring, data-driven anomaly detection and process-monitoring methods have been widely adopted in industrial systems, including support vector machines, autoencoders, recurrent neural networks, and Transformer-based architectures [11,12,13,14]. These approaches have substantially improved anomaly detection and predictive performance across a wide range of industrial applications.

Despite these advances, most existing studies remain prediction-oriented and primarily emphasize forecasting accuracy or classification performance. In many industrial applications, methane concentration is treated as an isolated prediction target, while the intermediate evolution process associated with abnormal events remains insufficiently characterized. In addition, existing event-based anomaly studies are often designed to identify abnormal segments or summarize event characteristics, but they rarely investigate how multisensor dependencies evolve within individual methane exceedance events. As a result, existing methods often provide limited interpretability regarding how multisensor abnormalities emerge, interact, and evolve prior to methane threshold exceedance.

To better capture system-level interactions, multivariate monitoring and multisensor fusion methods have increasingly been adopted in coal mine safety monitoring and gas-warning applications. Recent studies have integrated methane concentration, airflow velocity, temperature, dust concentration, and other environmental variables to improve hazard identification and safety early-warning performance through multi-source information fusion [9,15,16]. Multisensor fusion techniques provide effective ways to integrate heterogeneous monitoring signals and improve system-level situational awareness [17]. However, these approaches generally aggregate heterogeneous monitoring signals into unified representations and seldom explicitly characterize how interactions among variables evolve during individual methane exceedance events. Meanwhile, multivariate statistical monitoring methods, such as principal component analysis and latent-variable-based process monitoring, have been widely used to characterize abnormal operating conditions in industrial systems [3,18].

Recent studies have further explored graph-based monitoring frameworks and graph neural network models for coal mine safety monitoring and gas prediction. By representing monitoring variables as graph nodes and modeling their spatiotemporal interactions through graph structures, these approaches can effectively capture complex dependencies among heterogeneous monitoring signals [19,20,21]. Compared with isolated variable monitoring strategies, graph-based approaches provide richer system-level representations and improved predictive capability. However, the relationships among variables are typically embedded within graph-learning models and are primarily optimized for prediction tasks. Consequently, it remains difficult to directly interpret how abnormal multisensor evolution develops during individual methane exceedance events. Moreover, most graph-based approaches are designed to improve prediction accuracy or anomaly-detection performance, whereas event-level dependency evolution associated with methane exceedance remains insufficiently investigated.

Temporal dependency analysis and dynamic interaction modeling further provide theoretical tools for understanding relationships among monitoring variables in complex systems. Methods such as Granger causality, neural Granger causality, Bayesian networks, and transfer entropy have been widely applied to characterize directional dependencies and information transfer patterns in multivariate time-series systems [22,23,24,25,26,27,28,29]. These approaches provide useful theoretical foundations for analyzing lagged temporal relationships among industrial variables. However, most existing studies focus on global dependency structures or pairwise interactions and rarely provide event-specific and interpretable descriptions of abnormal evolution processes in industrial monitoring systems.

In recent years, interpretable artificial intelligence and explainable industrial monitoring have become increasingly important research directions in safety-critical systems [30]. Compared with purely black-box prediction models, industrial operators are often more concerned with understanding how abnormal conditions emerge and evolve across multiple subsystems before critical events occur. This trend highlights the need for interpretable event-driven analysis frameworks capable of characterizing multisensor abnormal evolution patterns from a system-level perspective.

Several limitations therefore remain in current methane monitoring studies. First, most existing approaches are designed for concentration prediction and do not explicitly characterize the temporal evolution process associated with methane exceedance events. Second, although multisensor fusion approaches improve warning performance by integrating heterogeneous monitoring signals, the interactions among variables are typically embedded implicitly within prediction models, limiting the interpretability of abnormal evolution processes. Third, existing graph-based monitoring and prediction approaches generally construct global dependency structures for model training and forecasting purposes, which may overlook the heterogeneity and event-specific characteristics of individual methane exceedance processes. Finally, although temporal dependencies among monitoring variables may exist, event-specific dependency structures and interpretable evolution paths are rarely analyzed explicitly.

## 2. Methodology

This section presents the proposed event-driven multisensor temporal dependency analysis framework for methane exceedance events in underground coal mines. The framework aims to characterize interpretable temporal evolution patterns associated with methane exceedance using multisensor monitoring data. Instead of modeling methane exceedance solely as a prediction problem, the proposed approach analyzes how multisensor abnormal patterns emerge and evolve prior to threshold exceedance from an event-driven perspective.

The proposed framework consists of five major components: methane exceedance event extraction, continuous risk representation, trigger detection, event-specific temporal dependency network construction, and cross-event evolution pattern analysis.

### 2.1. Problem Formulation

Coal mine monitoring systems consist of multiple heterogeneous sensors deployed in underground working areas to continuously record equipment operation, ventilation conditions, environmental parameters, methane drainage states, and methane concentration levels. These sensors generate multivariate time-series observations that reflect dynamic variations in underground mining systems.

Suppose that the monitoring system contains *n* sensors. The discrete-time system state at time *t* is represented as

(1) $$x\left(t\right)={\left[x_{1}\left(t\right),x_{2}\left(t\right),\ldots ,x_{n}\left(t\right)\right]}^{⊤},\;t=1,2,\ldots ,T$$

where $x_{i}\left(t\right)$ denotes the observation of sensor *i* at time *t*, and *T* is the total number of observations.

According to the sensor configuration of the public dataset, the monitored variables are organized into five groups:

- **Operational variables**, including cutter-loader current, haulage current, hydraulic pump current, working-face direction, and operating speed;
- **Ventilation variables**, including airflow velocity measurements in mine tunnels;
- **Environmental variables**, including temperature, humidity, and barometric pressure;
- **Methane drainage variables**, including drainage pressure, pipeline pressure, pipeline temperature, and methane flow conditions;
- **Methane response variables**, including methane concentration measurements at different monitoring locations.

Among all methane sensors, three target sensors are selected for exceedance analysis:

(2) $$M_{\mathrm{tar}}=\left\{MM263,MM264,MM256\right\}.$$

These sensors are equipped with predefined warning and alarm thresholds. Let $\delta_{W}$ and $\delta_{A}$ denote the methane warning and alarm thresholds, respectively:

(3) $$\delta_{W}=1.0\%,\;\delta_{A}=1.5\%.$$

A methane warning state is defined as

(4) $$Y_{W}\left(t\right)=\left\{\begin{array}{cc} 1,\; & \exists m\in M_{\mathrm{tar}},\;x_{m}\left(t\right)\geq \delta_{W},\\ 0,\; & \mathrm{otherwise}, \end{array}\right.$$

and a methane alarm state is defined as

(5) $$Y_{A}\left(t\right)=\left\{\begin{array}{cc} 1,\; & \exists m\in M_{\mathrm{tar}},\;x_{m}\left(t\right)\geq \delta_{A},\\ 0,\; & \mathrm{otherwise}. \end{array}\right.$$

In practical underground production environments, methane threshold exceedance generally does not occur instantaneously. Instead, methane-related abnormal events are often accompanied by progressive variations in operational conditions, ventilation states, environmental parameters, and methane drainage processes before the methane concentration reaches warning thresholds [2]. Therefore, the objective of this study is not merely to detect methane exceedance itself but to characterize the multisensor abnormal evolution process associated with methane exceedance events.

Given a methane exceedance event occurring at time $t_{e}$, the proposed framework aims to answer the following questions:

1. At what time does the monitoring system first exhibit sustained abnormal evolution before methane threshold exceedance?
2. How are lagged temporal dependencies among multisensor variables associated with methane exceedance events?
3. What are the representative temporal evolution paths associated with methane exceedance events?

Based on these considerations, the methane exceedance evolution analysis problem is formally defined as follows:

**Definition 1**  (Methane Exceedance Event Set)**.** *Let*

(6) $$E=\left\{t_{e}^{\left(1\right)},t_{e}^{\left(2\right)},\ldots ,t_{e}^{\left(N_{e}\right)}\right\}$$

*denote the set of independent methane exceedance events, where each $t_{e}^{\left(r\right)}$ is the first time point at which a target methane sensor reaches the warning or alarm threshold within an event segment.*

**Definition 2**  (Risk Trigger Point)**.** *For each methane exceedance event $t_{e}^{\left(r\right)}$, the trigger point $t_{r}^{*(r)}$ is defined as the earliest time instant before $t_{e}^{\left(r\right)}$ at which the monitoring system enters a persistent abnormal evolution state.*

**Definition 3**  (Event-Specific Temporal Dependency Network)**.** *For each event r, an event-specific temporal dependency network is defined as*

(7) $$G^{\left(r\right)}=\left(V,E^{\left(r\right)},W^{\left(r\right)},L^{\left(r\right)}\right),$$

*where V is the set of sensor nodes, $E^{\left(r\right)}\subseteq V\times V$ is the set of directed dependency edges in event r, $W^{\left(r\right)}=\left[w_{ij}^{\left(r\right)}\right]$ is the edge weight matrix, and $L^{\left(r\right)}=\left[\ell_{ij}^{\left(r\right)}\right]$ is the corresponding lag matrix.*

*The constructed network represents statistically inferred lagged temporal dependencies among monitoring variables within event windows, rather than direct physical methane transport mechanisms or strict causal relationships.*

**Definition 4**  (Temporal Evolution Dependency Path)**.** *Given the event-specific temporal dependency network $G^{\left(r\right)}$, a temporal evolution dependency path is defined as an ordered sequence of nodes*

(8) $$P^{\left(r\right)}=\left(v_{1},v_{2},\ldots ,v_{k}\right)$$

*such that*

(9) $$\left(v_{i},v_{i+1}\right)\in E^{\left(r\right)},\;i=1,\ldots ,k-1,$$

*and the terminal node satisfies*

(10) $$v_{k}\in M_{\mathrm{tar}}.$$

*Among all feasible paths $P^{\left(r\right)}$, the representative evolution dependency path is defined as*

(11) $$P^{*(r)}=\arg\max_{P\in P^{\left(r\right)}}{\mathrm{Score}}^{\left(r\right)}\left(P\right),$$

*where ${\mathrm{Score}}^{\left(r\right)}\left(P\right)$ measures the dependency strength and event-specific relevance of a candidate path.*

*Therefore, the overall objective of this study is to identify, for each methane exceedance event, the abnormal evolution trigger point, the event-specific temporal dependency structure, and representative evolution dependency paths associated with methane exceedance in underground coal mine systems.*

### 2.2. Framework of the Proposed Method

The proposed framework aims to characterize progressive abnormal evolution processes in complex multisensor industrial systems through event-oriented temporal dependency analysis and cross-event pattern discovery. Rather than treating abnormal events as isolated threshold exceedances, the framework models how multisensor interactions gradually evolve toward critical system states through persistent risk evolution.

**Figure 1:**
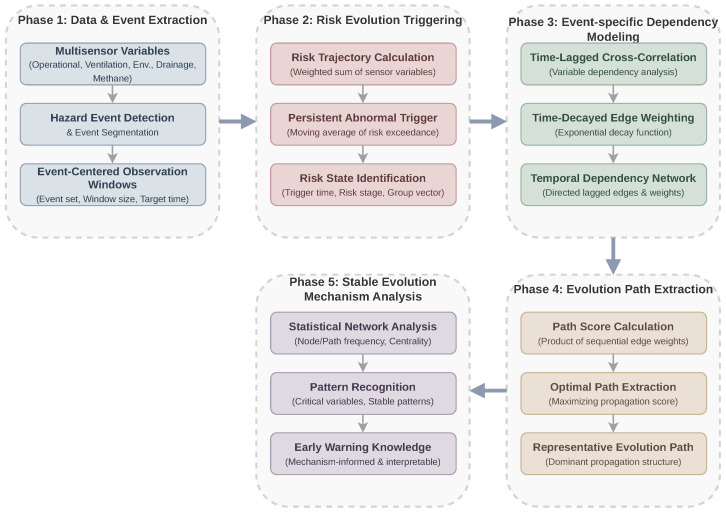
Framework of the proposed event-oriented multisensor risk-evolution analysis method.

As illustrated in Figure 1, the framework consists of five sequential phases, including event extraction, risk-evolution triggering, temporal dependency construction, representative evolution-path extraction, and cross-event pattern discovery. The overall framework progressively transforms raw multisensor monitoring signals into interpretable pattern-level early-warning knowledge through hierarchical abstraction of event dynamics, risk evolution, dependency development, and recurrent evolution structures. In the first phase, raw multisensor monitoring data containing operational, environmental, ventilation, drainage, and target-state variables are continuously collected. Critical industrial events are first identified through threshold-based event detection and temporal segmentation. For each detected event, an event-centered observation window is constructed to capture the pre-event evolution dynamics and surrounding contextual information. These event-centered windows serve as the fundamental analysis units for subsequent risk-evolution modeling.

In the second phase, dynamic risk trajectories are estimated from multisensor abnormality representations. A global risk indicator is constructed by aggregating sensor-level abnormal evolution signals, enabling continuous characterization of system-wide evolution intensity. Based on the temporal persistence of elevated risk states, a persistent abnormal evolution trigger is further identified to determine the earliest time at which the system enters a stable abnormal evolution process. The resulting trigger-aligned evolution state provides the temporal starting point for subsequent dependency analysis.

In the third phase, event-specific temporal dependency networks are constructed to characterize lagged multisensor interactions during abnormal evolution processes. Time-lagged statistical dependencies are estimated through cross-correlation analysis between sensor evolution trajectories, while temporal decay mechanisms are introduced to penalize excessively delayed interactions. The resulting directed dependency network represents the temporal dependency structure associated with abnormal evolution within each event.

In the fourth phase, representative evolution paths are extracted from the dependency network to identify dominant dependency structures associated with the target critical state. Path importance is evaluated according to cumulative dependency strength along sequential dependency edges, allowing the framework to identify interpretable evolution chains that characterize how abnormal interactions progressively develop across monitoring variables.

In the final phase, cross-event statistical analysis is performed to discover stable evolution patterns shared across multiple events. By aggregating recurrent nodes, dependency paths, edge frequencies, and network centrality patterns across event-specific dependency structures, the framework identifies critical precursor variables, stable recurrent dependency patterns, and representative abnormal evolution patterns. These recurrent structures provide interpretable pattern-level knowledge for industrial early warning and risk analysis.

### 2.3. Continuous Multisensor Risk Representation

To characterize the progressive abnormal evolution process associated with methane exceedance events, a continuous multisensor risk representation is constructed from heterogeneous monitoring variables.

Instead of directly relying on methane concentration alone, the proposed framework aggregates abnormal variations from multiple subsystems, including operational conditions, ventilation states, environmental parameters, methane drainage conditions, and methane-response variables, to form a system-level risk representation.

#### 2.3.1. Sensor-Level Anomaly Representation

Let $x_{i}\left(t\right)$ denote the observation of sensor *i* at time *t*. For each sensor, robust normalization is first applied using baseline statistics estimated from historical normal operating periods:

(12) $$z_{i}\left(t\right)=\frac{x_{i}\left(t\right)-\mu_{i}}{\sigma_{i}+\epsilon },$$

where $\mu_{i}$ and $\sigma_{i}$ denote the baseline median and median absolute deviation (MAD)-based scale estimate of sensor *i*, respectively, and ϵ is a small constant introduced for numerical stability.

Because heterogeneous monitoring variables may contribute differently to methane-related risk representation, a directional coefficient $s_{i}\in \left\{-1,+1\right\}$ is introduced.

The directional coefficient $s_{i}$ is introduced to provide a consistent risk-oriented interpretation of heterogeneous sensor anomalies. The assigned direction does not imply a universal causal or strictly monotonic relationship between an individual variable and methane risk under all operating conditions. Instead, it represents the expected risk orientation adopted for anomaly aggregation within the proposed multisensor risk-evolution framework.

Although sensors within the same subsystem share the same directional coefficient, the assignments are specified at the individual-sensor level. Sensors grouped in Table 1 receive identical coefficients because they represent the same physical quantity or operational characteristic and, therefore, share the same risk orientation within the proposed framework.

The complete sensor-level assignments are summarized in Table 1.

$s_{i}\in \left\{-1,+1\right\}$ is assigned as

(13) $$\phi_{i}\left(t\right)=\max\left(0,s_{i}z_{i}\left(t\right)\right),$$

where $\phi_{i}\left(t\right)$ denotes the directional anomaly score of sensor *i* at time *t*.

Positive values of $\phi_{i}\left(t\right)$ indicate abnormal variations associated with increasing methane-related system risk, whereas negative deviations are suppressed to avoid cancellation effects among heterogeneous variables.

#### 2.3.2. Subsystem-Level Risk Representation

To characterize coordinated abnormal evolution across different monitoring subsystems, sensors are grouped into operational, ventilation, environmental, methane-drainage, and methane-response categories.

For subsystem *g*, the subsystem-level risk is defined as

(14) $$q_{g}\left(t\right)=\sum_{i\in V_{g}}{\tilde{w}}_{i}\phi_{i}\left(t\right),$$

where $V_{g}$ denotes the set of sensors belonging to subsystem *g*, and ${\tilde{w}}_{i}$ is the normalized local importance weight of sensor *i* within the subsystem.

The subsystem-level representation allows the framework to capture coordinated abnormal evolution patterns among heterogeneous monitoring variables while preserving subsystem interpretability.

#### 2.3.3. Global Multisensor Risk Representation

Based on the sensor-level anomaly scores, the global multisensor risk is defined as

(15) $$R_{t}=\sum_{i=1}^{n}w_{i}\phi_{i}\left(t\right),$$

where $w_{i}$ denotes the global importance weight of sensor *i*, satisfying

(16) $$\sum_{i=1}^{n}w_{i}=1.$$

The resulting risk signal $R_{t}$ provides a continuous system-level representation of multisensor abnormal evolution intensity over time. In this study, equal weighting is adopted within each monitoring subsystem to provide a simple and interpretable aggregation of heterogeneous anomaly signals. The primary objective of the proposed framework is to characterize multisensor abnormal evolution patterns rather than to optimize sensor importance through additional weighting models. This design avoids introducing additional parameter estimation procedures and maintains a transparent risk representation. Compared with conventional methane-threshold monitoring, the proposed risk representation integrates precursor abnormalities from ventilation, environmental, drainage, operational, and methane-monitoring subsystems, thereby providing a continuous system-level description of evolving risk conditions before methane-threshold exceedance.

#### 2.3.4. Risk Stage Representation

To improve interpretability of abnormal evolution dynamics, the continuous risk signal is further converted into discrete evolution stages.

Let $\delta_{1}$ and $\delta_{2}$ denote two predefined risk thresholds satisfying

(17) $$0<\delta_{1}<\delta_{2}.$$

The thresholds are set to $\delta_{1}=0.30$ and $\delta_{2}=0.55$, corresponding to the early abnormal evolution stage and the high-risk evolution stage, respectively. The evolution stage variable $\eta_{t}$ is defined as

(18) $$\eta_{t}=\left\{\begin{array}{cc} 0,\; & R_{t}<\delta_{1},\\ 1,\; & \delta_{1}\leq R_{t}<\delta_{2},\\ 2,\; & R_{t}\geq \delta_{2},\\ 3,\; & \exists m\in M_{\mathrm{tar}},\;x_{m}\left(t\right)\geq \delta_{A}, \end{array}\right.$$

where

- $\eta_{t}=0$ denotes the normal operating stage;
- $\eta_{t}=1$ denotes the early abnormal evolution stage;
- $\eta_{t}=2$ denotes the high-risk evolution stage;
- $\eta_{t}=3$ denotes methane alarm or exceedance conditions.

This stage-based representation provides an interpretable description of how methane-related abnormal evolution progresses from weak multisensor deviations to critical methane exceedance conditions.

#### 2.3.5. Persistence-Based Trigger Detection

In practical underground monitoring environments, short-term fluctuations and transient disturbances frequently occur due to operational variability and sensor noise. To avoid unstable triggering caused by instantaneous fluctuations, persistent abnormal evolution is identified using a rolling-window trigger mechanism.

Given a persistence window of length *L*, the trigger indicator is defined as

(19) $$\Gamma \left(t\right)=\frac{1}{L}\sum_{k=0}^{L-1}I\left(R_{t-k}\geq \delta_{1}\right),$$

where I(·) is the indicator function.

A persistent abnormal evolution trigger is activated when

(20) Γ(t)≥τ,

where τ denotes the persistence ratio threshold.

The persistence window length is set to L=12, and the persistence ratio threshold is set to τ=0.50. Consequently, at least half of the observations within the rolling window must satisfy $R_{t}\geq \delta_{1}$ before a persistent abnormal evolution condition is identified.

The persistence parameters are introduced to suppress transient fluctuations commonly observed in underground monitoring systems. In particular, the choice of τ=0.50 provides a simple majority criterion that balances sensitivity to emerging abnormal evolution against robustness to short-duration disturbances.

These parameters were specified prior to benchmark evaluation and remained fixed throughout all experiments. No event-specific parameter tuning was performed.

The corresponding trigger point is defined as the earliest time instant satisfying the persistence criterion:

(21) $$t_{r}^{*}=\min\left\{t:\Gamma (t)\geq \tau \right\}.$$

Compared with instantaneous threshold alarms, the proposed persistence-based mechanism reduces sensitivity to isolated fluctuations and enables more stable identification of sustained abnormal evolution processes prior to methane exceedance.

### 2.4. Event-Specific Temporal Dependency Construction

To characterize interpretable temporal interactions among monitoring variables during methane exceedance evolution, event-specific temporal dependency networks are constructed within individual event windows.

Unlike global dependency modeling approaches applied to the entire monitoring period, the proposed framework focuses on event-centered short-term abnormal evolution processes. Because methane exceedance events exhibit strong heterogeneity in duration, subsystem participation, and precursor evolution intensity, global dependency structures may obscure localized event-specific interaction patterns. This strategy enables the framework to capture local multisensor interaction structures associated with individual methane exceedance events.

#### 2.4.1. Lagged Dependency Estimation

Pearson correlation is adopted because the objective of this study is to characterize short-term interpretable linear coordination patterns within event windows, rather than to establish strict nonlinear causality or global information-transfer structures. Although nonlinear dependencies may exist in complex underground systems, the present study focuses on interpretable event-specific coordination patterns and, therefore, adopts Pearson correlation as a simple and transparent dependency measure. For each event window *r*, lagged temporal dependencies between sensor variables are estimated using event-specific cross-correlation analysis.

Let $\phi_{i}\left(t\right)$ and $\phi_{j}\left(t\right)$ denote the directional anomaly scores of sensors *i* and *j*, respectively. For a candidate lag $\ell \in [0,\ell_{\max}]$, the lagged dependency coefficient is defined as

(22) $$\rho_{ij}^{\left(r\right)}\left(\ell \right)=\mathrm{Corr}\left(\phi_{i}\left(t-\ell \right),\phi_{j}\left(t\right)\right),$$

where Corr(·,·) denotes the Pearson correlation coefficient computed within the event window.

The optimal lagged dependency strength between sensors *i* and *j* is further defined as

(23) $$\rho_{ij}^{\left(r\right)}=\max_{\ell \in [0,\ell_{\max}]}\rho_{ij}^{\left(r\right)}\left(\ell \right),$$

and the corresponding optimal lag is

(24) $$\ell_{ij}^{\left(r\right)}=\arg\max_{\ell \in [0,\ell_{\max}]}\rho_{ij}^{\left(r\right)}\left(\ell \right).$$

Therefore, $\rho_{ij}^{\left(r\right)}$ represents the maximum event-specific lagged dependency strength between sensors *i* and *j*, while $\ell_{ij}^{\left(r\right)}$ denotes the corresponding temporal delay.

Compared with instantaneous correlation analysis, the proposed lagged dependency estimation allows short-term temporal dependency patterns among heterogeneous monitoring variables to be captured.

#### 2.4.2. Dependency Edge Construction

To suppress weak and unstable interactions, only statistically meaningful dependencies are retained in the temporal dependency network.

A directed edge from node *i* to node *j* is established when

(25) $$\rho_{ij}^{\left(r\right)}\geq \theta ,$$

where θ is the dependency threshold. In this study, θ=0.20 is adopted to suppress weak associations while preserving the dominant event-specific dependency structure.

The dependency edge weight is further defined as

(26) $$w_{ij}^{\left(r\right)}=\rho_{ij}^{\left(r\right)}\exp\left(-\beta \ell_{ij}^{\left(r\right)}\right),$$

where β>0 is the lag-penalty coefficient. In this study, β=0.20 is adopted to provide moderate suppression of excessively long-lag dependencies while retaining interpretable short-term temporal associations.

The exponential penalty term suppresses excessively long-range dependencies and encourages compact short-term temporal interaction structures.

The resulting event-specific temporal dependency network is therefore defined as

(27) $$G^{\left(r\right)}=\left(V,E^{\left(r\right)},W^{\left(r\right)},L^{\left(r\right)}\right),$$

where

- *V* denotes the set of monitoring sensors;
- $E^{\left(r\right)}$ denotes the set of retained dependency edges;
- $W^{\left(r\right)}=\left[w_{ij}^{\left(r\right)}\right]$ denotes the dependency weight matrix;
- $L^{\left(r\right)}=\left[\ell_{ij}^{\left(r\right)}\right]$ denotes the lag matrix.

#### 2.4.3. Interpretation of Dependency Structures

The constructed dependency networks represent statistically inferred lagged temporal associations among multisensor abnormal signals within methane exceedance events.

It should be emphasized that the proposed framework does not aim to establish strict physical causality or methane transport mechanisms. Therefore, the resulting dependency structures should be interpreted as statistical association patterns rather than evidence of direct physical interactions or causal relationships. Instead, the inferred dependencies describe event-specific temporal coordination patterns among operational, ventilation, environmental, methane-drainage, and methane-response variables during abnormal evolution processes.

Compared with global dependency analysis, the proposed event-specific construction strategy provides improved interpretability for understanding how multisensor abnormalities progressively evolve before methane exceedance. In addition, the inferred lagged dependencies may be influenced by common trends, temporal autocorrelation, shared operational activities, and other latent factors that are not explicitly modeled in the present framework. Consequently, the constructed dependency networks should be interpreted as event-specific statistical association structures rather than direct physical interaction pathways or causal mechanisms. The identified temporal dependencies are intended to support interpretable characterization of abnormal evolution patterns and should not be regarded as evidence of physical methane transport processes or strict subsystem causality.

### 2.5. Representative Evolution Path Identification

Based on the event-specific temporal dependency network, representative evolution paths are extracted to describe how multisensor abnormalities are temporally associated with the target methane sensor.

For each event-specific network $G^{\left(r\right)}$, a feasible temporal dependency path is defined as an ordered node sequence

(28) $$P^{\left(r\right)}=\left(v_{1},v_{2},\ldots ,v_{K}\right),$$

where

(29) $$\left(v_{k},v_{k+1}\right)\in E^{\left(r\right)},\;k=1,\ldots ,K-1,$$

and the terminal node is the target methane sensor:

(30) $$v_{K}=m,\;m\in M_{\mathrm{tar}}.$$

To quantify the strength of a candidate path, the path score is defined as the product of edge weights along the path:

(31) $${\mathrm{Score}}^{\left(r\right)}\left(P\right)=\prod_{k=1}^{K-1}w_{v_{k}v_{k+1}}^{\left(r\right)}.$$

This multiplicative scoring strategy requires all dependency links along the path to be consistently strong. If any edge in the path has weak dependency strength, the overall path score will decrease accordingly. Therefore, the selected path is not simply the longest path or the path containing the largest number of edges, but the strongest temporally connected dependency chain leading to the methane target. The multiplicative formulation intentionally favors dependency chains with consistently strong associations across all intermediate links. Consequently, paths containing weak intermediate dependencies are naturally down-weighted, improving the interpretability and reliability of the extracted representative evolution path.

The representative temporal evolution path for event *r* is then defined as

(32) $$P^{*(r)}=\arg\max_{P\in P^{\left(r\right)}}{\mathrm{Score}}^{\left(r\right)}\left(P\right),$$

where $P^{\left(r\right)}$ denotes the set of feasible directed paths ending at the target methane sensor within the event-specific dependency network.

To avoid excessively long and weakly interpretable dependency chains, the maximum path depth is constrained by a predefined parameter $D_{\max}$. In this study, $D_{\max}=4$ is adopted to balance path interpretability and dependency coverage. Longer paths are more likely to contain indirect associations and are generally more difficult to interpret from an operational perspective. In addition, because the path score is computed as the product of edge weights, longer paths are naturally penalized. Only paths satisfying

(33) $$K\leq D_{\max}$$

are considered during path searching.

The corresponding maximum path intensity is denoted as

(34) $$P_{t}=\max_{P\in P^{\left(r\right)}}{\mathrm{Score}}^{\left(r\right)}\left(P\right),$$

which reflects the strongest dependency-chain intensity directed toward the methane target within the local event window.

In implementation, the representative path is identified through depth-limited backward search from the target methane sensor. At each step, preceding nodes with positive dependency weights are recursively explored, and the path with the highest multiplicative score is retained.

The extracted representative path provides an interpretable description of the dominant multisensor dependency sequence associated with methane exceedance evolution.

### 2.6. Model Assumptions

To formulate methane exceedance evolution analysis as a tractable multisensor time-series problem, the following assumptions are introduced:

**Assumption 1**  (Discrete sampling)**.** *The monitoring system operates under a fixed discrete sampling interval, and multisensor observations are synchronized in time. Discrete synchronized sampling is commonly adopted in industrial process monitoring and multivariate time-series analysis [3,5,31].*

**Assumption 2**  (Progressive abnormal evolution)**.** *Methane warning or alarm events are generally associated with progressive abnormal evolution processes involving coupled multisensor variations before threshold exceedance. Progressive abnormal evolution and anomaly accumulation have been widely discussed in industrial anomaly analysis and system safety research, where critical events are often preceded by gradual deviations across interconnected subsystems [2,4,13,32].*

**Assumption 3**  (Time-lagged dependency)**.** *Temporal interactions among monitoring variables are not instantaneous. Sensor observations may exhibit lagged statistical dependencies with temporally preceding variables:*

(35) $$x_{j}\left(t\right)=f_{j}\left(x_{1}\left(t-\ell_{1j}\right),x_{2}\left(t-\ell_{2j}\right),\ldots ,x_{n}\left(t-\ell_{nj}\right)\right)+\varepsilon_{j}\left(t\right),$$

*where $\ell_{ij}\geq 0$ is the lag from node i to node j, and $\varepsilon_{j}\left(t\right)$ is a stochastic disturbance term.*

*Lagged temporal dependency modeling has been widely adopted in dynamic system analysis, industrial monitoring, and time-series dependency inference [5,22,23,24,29].*

**Assumption 4**  (Local structural stability)**.** *Within a short time window before methane exceedance, dominant dependency structures among monitoring variables remain approximately stable, which makes event-specific dependency analysis feasible. Local structural stability assumptions are commonly employed in short-window process monitoring and change-point analysis for nonstationary industrial systems [33,34,35].*

**Assumption 5**  (Structured multi-subsystem evolution)**.** *Methane exceedance events are generally associated with coordinated variations across operational, ventilation, environmental, drainage, and methane-related subsystems rather than isolated fluctuations of a single variable. Coordinated multi-subsystem abnormal evolution has been widely recognized in complex industrial systems, multisensor monitoring, and interdependent system analysis [17,30,32].*

### 2.7. Interpretation of Temporal Dependency Networks

The proposed temporal dependency networks are designed to provide interpretable representations of multisensor abnormal evolution patterns within methane exceedance events.

In the constructed networks, nodes correspond to monitoring sensors deployed in underground coal mine systems, while directed edges represent statistically significant lagged temporal dependencies inferred within event windows.

The direction of an edge reflects temporal precedence between monitoring variables rather than physical methane transport direction. Therefore, the constructed dependency networks should not be interpreted as physical gas-flow networks or strict causal graphs.

Instead, the proposed framework provides event-specific representations of how multisensor abnormalities are temporally associated during methane exceedance evolution processes. This event-driven dependency analysis complements existing prediction-oriented methods by improving interpretability of multisensor abnormal evolution patterns.

## 3. Dataset and Experimental Setup

### 3.1. Dataset Description

The analysis was conducted using a publicly available underground coal mine monitoring dataset from the Mendeley Data repository (https://data.mendeley.com/datasets/yd7vw4c5mk/1, accessed on 3 March 2026). The dataset was collected from an underground monitoring system designed to continuously record ventilation conditions, environmental variables, methane concentration, methane drainage status, and mining equipment operation.

The monitoring system records multivariate sensor observations at a sampling interval of one second. Each record contains timestamp information together with measurements from multiple sensors deployed across different parts of the mining system, capturing dynamic interactions among operational activities, ventilation conditions, environmental states, methane drainage processes, and methane concentration evolution.

Unlike laboratory-scale datasets with controlled conditions, the monitoring data were collected under real underground operating environments, where mining activities, airflow conditions, and methane emissions vary continuously over time. As a result, the dataset exhibits strong temporal fluctuations, nonstationarity, and heterogeneous subsystem interactions, making it suitable for studying event-driven abnormal evolution patterns in complex industrial monitoring systems.

Table 2 summarizes the sensors included in the dataset.

### 3.2. Target Sensors and Event Definition

Among the methane sensors, MM263, MM264, and MM256 were selected as target sensors because they are located close to active mining areas, where methane accumulation is more likely to occur.

**Figure 2:**
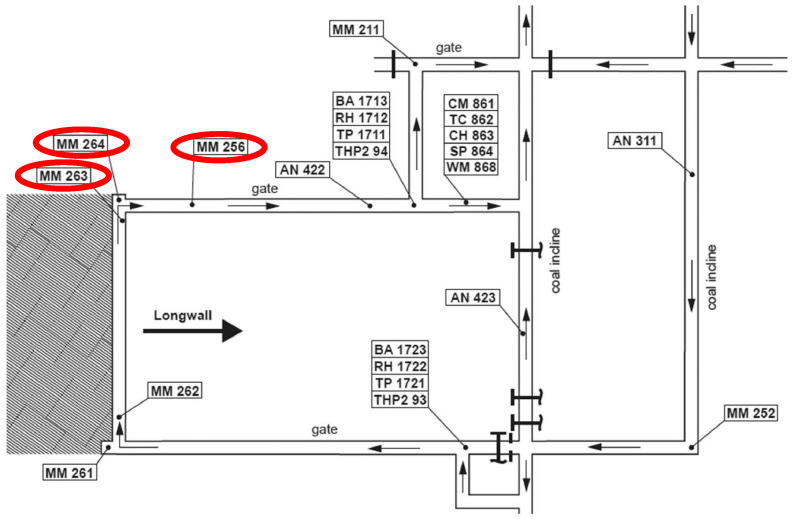
Spatial layout of the underground monitoring system and locations of the selected target methane sensors. Adapted from the original dataset publication [36].

The spatial layout of the underground monitoring system is illustrated in Figure 2. The selected target methane sensors are located in the monitored longwall production area and are directly associated with methane exceedance monitoring.

According to the monitoring configuration, methane safety thresholds are defined as follows:

(36) $$\delta_{W}=1.0\%\;{\mathrm{CH}}_{4},\;\delta_{A}=1.5\%\;{\mathrm{CH}}_{4}.$$

Methane exceedance events are identified based on threshold crossings of the target sensors. An event is defined as a continuous time interval during which methane concentration exceeds the predefined threshold.

To avoid fragmentation caused by short-term fluctuations around the threshold, temporally adjacent exceedance segments are merged when the gap between consecutive exceedances is smaller than 300 s. This setting is intended to prevent a single evolving methane accumulation process from being artificially divided into multiple independent events due to brief threshold recoveries. For each detected event, an event-centered observation window is constructed to capture the precursor phase, methane exceedance phase, and post-event recovery process. A 600 s precursor observation interval before the detected event-segment start is adopted to capture sufficient multisensor evolution information while avoiding excessive inclusion of unrelated normal operating periods.

It should be noted that the detected event-segment start and the first methane-warning occurrence are not necessarily identical. Therefore, the 600 s precursor interval is used to define the event-analysis window for trigger identification and dependency analysis, rather than the maximum possible warning lead time.

The event window consists of the following:

- A 600 s precursor observation interval before the detected event-segment start;
- The methane exceedance segment itself;
- A post-event recovery interval of 180 s after the methane concentration returns below the threshold.

All event-extraction parameters were fixed prior to benchmark evaluation and remained unchanged throughout all experiments. No event-specific parameter tuning was performed.

Table 3 summarizes the statistical characteristics of the extracted events.

The predominance of warning events suggests that methane accumulation typically evolves gradually before reaching critical alarm conditions. Furthermore, the existence of temporally extended events indicates that methane exceedance is often associated with structured abnormal evolution processes rather than isolated instantaneous spikes.

### 3.3. Data Preprocessing

Prior to risk-evolution analysis, several preprocessing procedures were applied to improve temporal consistency and reduce the influence of noise.

First, missing values were handled using forward filling for short gaps. Second, all sensor variables were normalized independently using robust statistics to reduce the influence of extreme outliers:

(37) $$z_{i}\left(t\right)=\frac{x_{i}\left(t\right)-\mathrm{median}\left(x_{i}\right)}{\mathrm{MAD}(x_{i})+\epsilon },$$

where MAD(·) denotes the median absolute deviation and ϵ is a small constant for numerical stability. The normalization statistics were estimated from an initial baseline segment of the monitoring data and subsequently applied throughout the analysis. Therefore, future observations contained in later event periods were not used when constructing the normalization reference, avoiding information leakage during risk estimation. Compared with conventional mean–standard deviation normalization, robust normalization provides better stability under highly fluctuating industrial monitoring conditions [37].

After normalization, directional anomaly transformation was applied according to the physical meaning of each variable category. Variables positively associated with methane accumulation retained their original sign, while variables negatively associated with methane risk were sign-adjusted to maintain consistent abnormality interpretation across subsystems.

### 3.4. Experimental Parameters

The proposed framework contains several parameters associated with persistent trigger detection, risk-stage characterization, and event-specific temporal dependency analysis. All parameters were selected to balance early-warning sensitivity, robustness against transient fluctuations, and interpretability of multisensor evolution structures under noisy underground monitoring environments.

#### 3.4.1. Trigger Detection Parameters

To identify sustained abnormal evolution rather than isolated instantaneous fluctuations, a persistence-based trigger mechanism is adopted.

The persistence window length is set to

(38) L=12,

corresponding to 12 s under the one-second sampling interval.

In underground monitoring environments, short-term signal oscillations frequently occur due to equipment switching, ventilation disturbances, operational variability, and sensor noise. If trigger activation is directly based on instantaneous abnormal observations, the monitoring system may generate unstable and noisy alarms.

Therefore, a short rolling persistence window is introduced to suppress transient fluctuations while still preserving precursor dynamics before methane exceedance. The selected window length remains substantially shorter than the duration of sustained methane-evolution processes observed in the dataset and, therefore, does not significantly delay early-warning activation.

The persistence ratio threshold is defined as

(39) τ=0.5,

meaning that abnormal evolution must persist during at least half of the observations within the rolling window before trigger activation.

This majority-based persistence criterion improves trigger stability and reduces false alarms caused by isolated abnormal spikes.

#### 3.4.2. Risk Stage Parameters

To characterize different abnormal evolution intensities, two stage-transition thresholds were introduced:

(40) $$\delta_{1}=0.30,\;\delta_{2}=0.55.$$

These thresholds divide the continuous multisensor risk signal into normal, early abnormal, and high-risk evolution stages. The lower threshold $\delta_{1}$ is designed to identify weak but persistent multisensor precursor deviations, while the higher threshold $\delta_{2}$ characterizes coordinated high-risk abnormal evolution before methane exceedance.

#### 3.4.3. Temporal Dependency Parameters

For event-specific temporal dependency analysis, the maximum lag length is defined as

(41) $$\ell_{\max}=5,$$

allowing short-term lagged temporal interactions among monitoring variables to be captured.

Because methane-evolution processes in underground monitoring systems are typically associated with local short-term subsystem interactions, excessively large lag windows may introduce unstable or weakly interpretable long-range dependencies. Therefore, the lag range is restricted to preserve local temporal interaction structures.

To suppress weak and noisy dependencies, the dependency edge threshold is defined as

(42) θ=0.20.

Only dependency relationships satisfying

(43) $$\rho_{ij}^{\left(r\right)}\geq \theta$$

are retained in the event-specific temporal dependency networks.

To further suppress excessively delayed interactions, lag-aware edge weighting is introduced:

(44) $$w_{ij}^{\left(r\right)}=\rho_{ij}^{\left(r\right)}\exp\left(-\beta \ell_{ij}^{\left(r\right)}\right),$$

where

(45) β=0.20

controls the attenuation of dependency strength with increasing temporal lag.

This exponential lag penalty encourages compact and temporally interpretable dependency structures while reducing the influence of excessively long-range delayed interactions.

### 3.5. Baseline Methods

To comprehensively evaluate the proposed interpretable multisensor risk-evolution framework, multiple representative benchmark methods from industrial monitoring, statistical process monitoring, unsupervised anomaly detection, and temporal change detection are considered.

#### 3.5.1. Statistical Process Monitoring Baselines

Traditional industrial monitoring methods are adopted to represent classical statistical deviation-detection strategies widely used in process monitoring systems.

The evaluated statistical process-monitoring baselines include the following:

- Shewhart control charts;
- Cumulative sum (CUSUM) monitoring;
- PCA-T^2^ statistics;
- PCA-SPE statistics.

These methods primarily identify abnormal operating conditions through statistical deviations from baseline operating distributions.

#### 3.5.2. Unsupervised Anomaly Detection Baselines

Representative unsupervised anomaly-detection methods are further included to characterize data-driven abnormality detection without requiring labeled fault samples.

The evaluated unsupervised baselines include the following:

- One-Class SVM;
- Isolation Forest;
- Local Outlier Factor (LOF);
- KMeans-distance anomaly scoring.

These methods generate continuous anomaly scores based on distributional deviation, local density variation, clustering distance, or boundary-based outlier characterization.

#### 3.5.3. Dynamic Change Baseline

A rate-of-change (ROC) detector is additionally included to represent simple temporal-gradient-based warning strategies. Unlike distribution-based anomaly detectors, the ROC baseline focuses on abrupt short-term signal variation intensity.

#### 3.5.4. Unified Trigger Conversion and Fair Comparison

Most benchmark methods considered in this study naturally produce continuous anomaly scores rather than discrete early-warning triggers. To enable fair comparison under a unified early-warning setting, all benchmark methods are converted into the same trigger-based evaluation framework.

For each method, continuous anomaly scores are first computed over the event windows. A percentile-based thresholding strategy is then applied to convert anomaly scores into binary abnormal states.

To avoid introducing method-specific evaluation bias, the same threshold-selection strategy is adopted across all benchmark methods, and no manual threshold tuning is performed for individual methods.

After thresholding, all benchmark methods are further converted into trigger sequences using the same persistence-based trigger mechanism adopted in the proposed framework. Specifically, a trigger is activated only when abnormal states persist within a rolling window according to the predefined persistence criterion.

Therefore, all methods are evaluated under identical persistence rules, trigger definitions, and lead-time evaluation criteria.

Under this unified benchmark setting, differences in early-warning performance mainly reflect the capability of different methods to characterize stable multisensor precursor evolution patterns before methane exceedance, rather than differences in trigger engineering strategies.

Compared with these baselines, the proposed framework explicitly models continuous multisensor risk evolution, subsystem interactions, temporal dependency development, critical-node activation, and representative evolution paths, thereby providing interpretable early-warning capability rather than purely reactive anomaly detection.

## 4. Results and Discussion

### 4.1. Overall Event Characteristics

**Figure 3:**
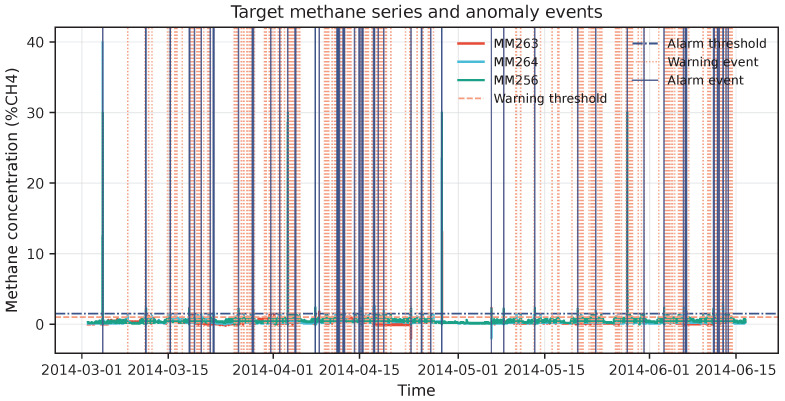
Time series of methane concentration for target sensors.

Figure 3 shows the methane concentration series of the three target sensors together with the warning and alarm thresholds.

The monitoring data exhibit strong temporal fluctuations and significant heterogeneity across different sensors, reflecting the dynamic nature of underground mining environments. Methane exceedance events are unevenly distributed over time and exhibit varying durations and intensities.

**Figure 4:**
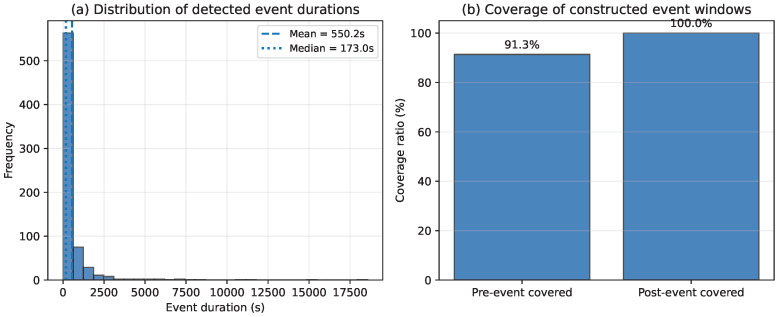
Validation of event detection and window construction. (**a**) Distribution of detected event durations. (**b**) Coverage ratios of pre-event and post-event intervals in the constructed windows.

Figure 4a further presents the distribution of event durations. Most detected events persist over meaningful temporal intervals rather than appearing as isolated spikes, indicating that methane exceedance is generally associated with temporally extended abnormal evolution processes.

Figure 4b shows that the constructed event windows maintain high coverage for both precursor and recovery stages, ensuring that the evolution process before methane exceedance is sufficiently captured.

These observations indicate that the extracted event windows provide a reliable basis for subsequent analysis of abnormal evolution patterns and temporal dependency structures.

### 4.2. Early Warning Capability

To evaluate whether the proposed framework can provide operationally useful precursor information before methane exceedance, benchmark experiments were conducted against multiple statistical process-monitoring and anomaly-detection baselines.

**Figure 5:**
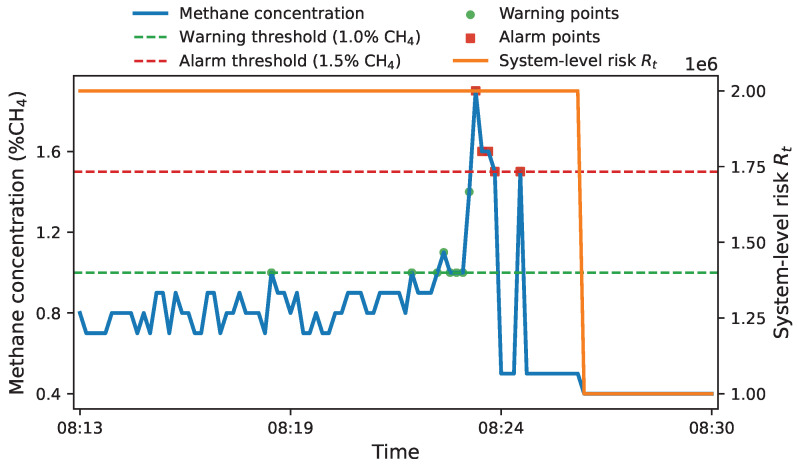
Temporal evolution of methane concentration and system-level risk in a representative event window.

Figure 5 presents a representative methane exceedance event window for illustrative interpretation.

The proposed system-level risk signal $R_{t}$ begins to increase before the methane concentration reaches the warning threshold, indicating that multisensor precursor deviations emerge prior to observable methane exceedance.

Although individual events exhibit heterogeneous temporal dynamics, this example illustrates the typical abnormal evolution behavior characterized by the proposed framework.

To quantitatively evaluate early-warning performance, trigger-time-based benchmark experiments were performed across all event windows.

Unlike conventional reactive alarms, an event is considered to have been successfully predicted only when the corresponding trigger occurs at least 60 s before the methane concentration exceeds the warning threshold.

This criterion was introduced to distinguish true precursor warning from synchronous threshold alarms.

The lead time is defined as the temporal difference between the first methane-threshold crossing time and the earliest persistent trigger time:

(46) $$\mathrm{LeadTime}=t_{\mathrm{warning}}-t_{r}^{*},$$

where $t_{\mathrm{warning}}$ denotes the first methane-warning occurrence and $t_{r}^{*}$ denotes the earliest persistent trigger identified by the proposed framework.

Therefore, the reported effective warning rates primarily reflect stable precursor detectability rather than simple alarm sensitivity.

**Figure 6:**
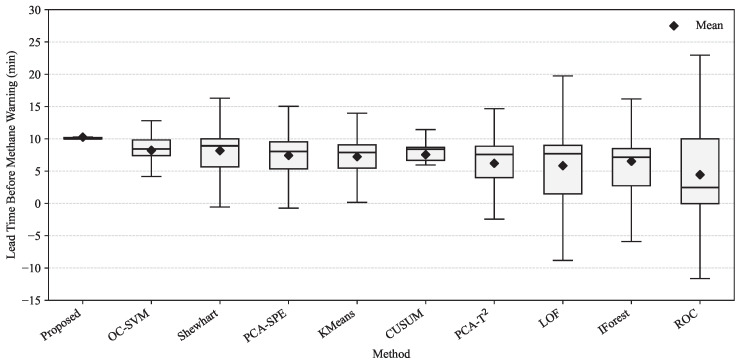
Distribution of effective lead times across benchmark methods.

Figure 6 compares the distributions of effective lead times across different benchmark methods.

The proposed framework exhibits both the longest median effective lead time and a relatively concentrated distribution compared with most baseline methods, indicating that the proposed multisensor risk-evolution framework provides more stable precursor warning capability.

Several statistical process-monitoring and unsupervised anomaly-detection methods also produce positive effective lead times. However, their distributions exhibit larger variability and shorter median lead times, suggesting less stable precursor detection performance.

The rate-of-change baseline occasionally produces extremely early triggers, resulting in relatively large lead times for a small subset of events. Nevertheless, its effective early-warning rate remains substantially lower than that of the proposed framework, indicating poor robustness and temporal consistency.

Table 4 summarizes the benchmark comparison results.

To provide a more comprehensive evaluation of early-warning capability, both system-level and sensor-specific warning performances are reported.

The benchmark evaluation was conducted on the same set of extracted event windows. Under the Any-Target-Sensor criterion, an event window is considered to have been successfully warned if a valid warning is generated before the earliest methane-warning occurrence observed among the three target methane sensors (MM263, MM264, or MM256) within that window.

For sensor-specific evaluation, warning performance was calculated separately for each target methane sensor. Because a single event window may contain methane-warning occurrences at more than one target sensor, the numbers of warning windows associated with MM256, MM263, and MM264 are not mutually exclusive and, therefore, do not sum to the total number of evaluated event windows.

To further clarify the difference between the Any-Target-Sensor and sensor-specific evaluations, Table 5 summarizes the warning occurrences and effective warning results for each target methane sensor.

Among the evaluated event windows, methane-warning occurrences were observed in 338, 99, and 297 windows for MM256, MM263, and MM264, respectively. The corresponding effective warning counts were 292, 92, and 258. When conditioned on windows in which the corresponding sensor actually reached the warning threshold, the effective warning rates were 0.864, 0.929, and 0.869, respectively.

These results indicate that the lower MM263-specific effective warning rate reported in Table 4 primarily reflects the relatively small number of MM263 warning occurrences under the all-window evaluation denominator, rather than substantially weaker warning performance. In contrast, the Any-Target-Sensor criterion aggregates warning occurrences across all three target sensors and, therefore, evaluates whether the proposed framework can provide a valid precursor warning somewhere within the monitored target region before methane-threshold exceedance. Together, these two evaluation perspectives provide complementary views of practical early-warning capability. It should be noted that the mean and median effective lead times reported in Table 4 correspond to the MM263-specific evaluation. MM263 was selected as the reference target sensor for detailed lead-time comparison with benchmark methods, whereas the Any-Target-Sensor criterion was introduced as a complementary system-level evaluation metric. The benchmark results indicate that the proposed framework achieves the best overall warning performance under both evaluation criteria. Under the Any-Target-Sensor criterion, the proposed framework achieves a raw detection rate of 0.925 and an effective warning rate of 0.848, both higher than those of the benchmark methods. For the MM263-specific evaluation, the corresponding raw detection rate and effective warning rate are 0.165 and 0.154, respectively.

It should be noted that the raw detection rate and the effective early-warning rate represent different evaluation criteria. The raw detection rate evaluates whether a method produces a trigger before or at methane-threshold exceedance, whereas the effective early-warning rate additionally requires the trigger to occur at least 60 s before methane-threshold exceedance.

Furthermore, the effective early-warning criterion adopted in this study is intentionally strict because a valid warning must simultaneously satisfy both lead-time requirements and persistence constraints under highly noisy underground monitoring conditions. Therefore, the reported effective warning rates primarily reflect stable precursor detectability rather than simple alarm sensitivity.

To further examine warning-state persistence, Table 6 summarizes the occupancy characteristics of warning and alarm states. The combined warning-or-alarm occupancy ratio is only 2.36%, indicating that the proposed framework does not remain continuously in an alert state and primarily generates localized warning responses during abnormal evolution periods.

The benchmark results also reveal noticeable differences between the two evaluation perspectives. Both evaluation criteria were computed on the same set of evaluated event windows. However, the Any-Target-Sensor criterion considers successful warning at any of the three target methane sensors (MM263, MM264, or MM256), whereas the MM263 criterion only considers warnings associated with MM263. Consequently, higher warning rates are expected under the Any-Target-Sensor evaluation because it additionally captures events whose precursor abnormalities are more evident at MM264 or MM256. In general, warning rates under the Any-Target-Sensor criterion are substantially higher than those obtained from the MM263-specific evaluation. This observation suggests that abnormal evolution patterns associated with methane exceedance are often detectable somewhere within the monitored target region before they become sufficiently pronounced at a specific methane sensor.

Under the MM263-specific evaluation, the proposed framework achieves the highest effective warning rate among all compared methods while simultaneously maintaining the longest effective lead time. The mean effective lead time reaches 660.8 s, and the median effective lead time reaches 600 s, indicating that the proposed multisensor risk-evolution representation can identify relatively stable precursor abnormal patterns substantially earlier than methane-threshold exceedance. It should be noted that the 600 s precursor interval defines the event-analysis window used for trigger identification and dependency analysis, rather than the maximum possible lead time. The lead time is calculated as the temporal difference between the first methane-warning occurrence and the earliest persistent trigger time.

The precursor interval is constructed relative to the detected event-segment start. Because lead time is calculated relative to the first methane-warning occurrence, whereas the precursor interval is defined relative to the detected event-segment start, the reported effective lead time is not constrained by the predefined 600 s precursor interval.

Therefore, the reported lead times should not be interpreted as being bounded by the 600 s precursor observation interval. To further evaluate whether the observed improvement is statistically significant, event-level McNemar tests were conducted under the Any-Target-Sensor evaluation criterion using the 600 s pre-event window. As shown in Table 7, the proposed framework significantly outperformed all benchmark methods (p<0.001 for all comparisons). In particular, compared with Shewhart-Z, the proposed framework successfully generated effective warnings in 44 additional events, whereas no events were successfully detected by Shewhart-Z but missed by the proposed framework. These results indicate that the observed performance improvement is statistically significant rather than arising from random variation.

Traditional statistical process-monitoring methods such as Shewhart-Z, CUSUM, PCA-T^2^, and PCA-SPE also produce moderate early-warning capability because they can capture distributional deviations and multivariate operating-state changes. However, these approaches primarily rely on global statistical deviation characterization and, therefore, provide less stable precursor identification under highly heterogeneous event conditions.

Representative unsupervised anomaly-detection methods, including One-Class SVM, Isolation Forest, LOF, and KMeans-distance scoring, achieve comparable but generally lower effective warning performance. Although these methods can identify abnormal operating regions in high-dimensional monitoring space, they do not explicitly model progressive multisensor evolution structures or lagged subsystem interactions associated with methane exceedance processes.

The rate-of-change baseline exhibits the largest mean lead time among all benchmark methods. However, its effective warning rate remains substantially lower than that of the proposed framework, indicating that abrupt local fluctuations occasionally trigger extremely early alarms but lack stable precursor consistency across events. This observation further suggests that methane exceedance evolution is not solely characterized by instantaneous signal variation intensity but is more strongly associated with persistent multisensor abnormal evolution processes involving coordinated subsystem interactions.

### 4.3. Event Structure and Stage Characteristics

To further characterize the internal structure of methane exceedance events, the distribution of stage durations was analyzed.

**Figure 7:**
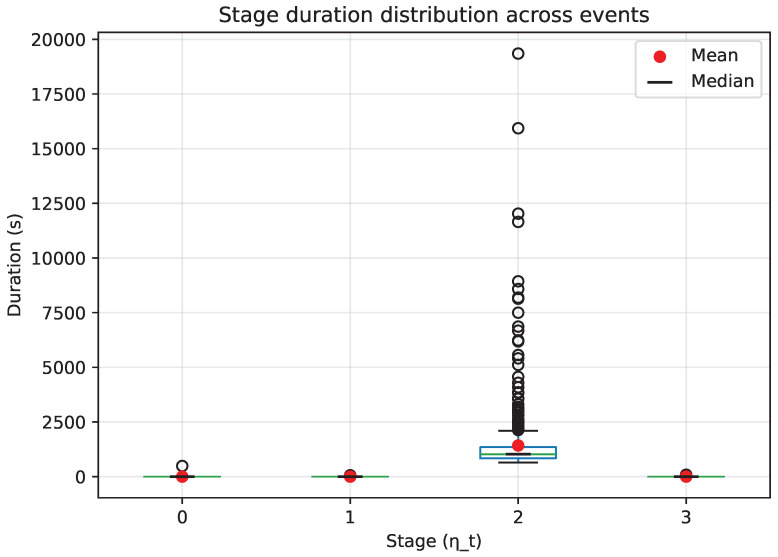
Distribution of stage durations across all event windows.

Figure 7 presents the duration distribution for each stage $\eta_{t}\in \left\{0,1,2,3\right\}$. The results show that stage $\eta_{t}=2$ dominates the evolution process, exhibiting both the largest duration and the highest variability across events.

In contrast, stages $\eta_{t}=0$, $\eta_{t}=1$, and $\eta_{t}=3$ are relatively short-lived. The limited duration of stage $\eta_{t}=1$ suggests that the precursor phase is temporally compressed, with rapid transitions from initial deviations to higher risk levels. Stage $\eta_{t}=3$, corresponding to threshold exceedance, appears only briefly, indicating that it represents a critical transition point rather than a sustained state.

These results indicate that methane risk evolution is primarily characterized by a dominant intermediate regime, in which the system remains in a high-risk yet pre-critical state for a substantial period.

### 4.4. Temporal Dependency Structure Analysis

To characterize the dependency structure of methane evolution, event-specific temporal dependency networks were constructed for all detected events.

**Figure 8:**
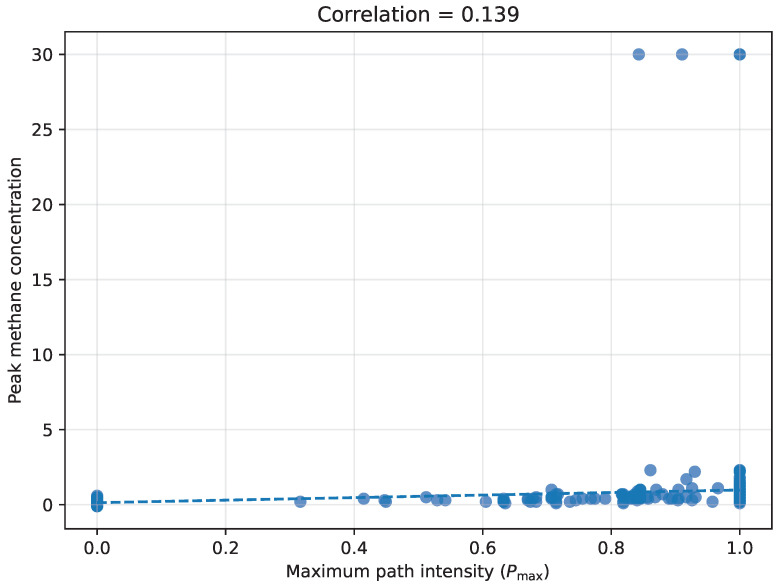
Relationship between maximum path intensity $P_{\max}$ and peak methane concentration across event windows.

Figure 8 shows the relationship between the maximum path intensity $P_{\max}$ and the peak methane concentration across all events.

A weak positive association can be observed between $P_{\max}$ and peak methane concentration. Given the relatively small correlation coefficient, this result should not be interpreted as evidence of a robust predictive relationship between evolution-path intensity and methane exceedance severity. Instead, it serves as supplementary and illustrative evidence that stronger temporal dependency structures may coexist with more severe methane exceedance processes in some events.

However, considerable heterogeneity exists across events, which may partially explain the relatively weak overall correlation. Some events exhibit highly structured dependency patterns involving multiple subsystems, whereas others remain dominated by relatively local methane dynamics.

**Figure 9:**
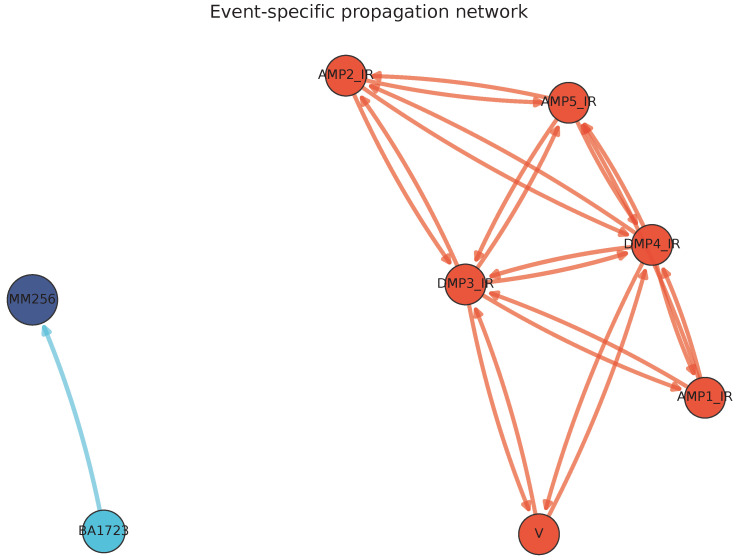
Event-specific temporal dependency network.

To further examine the dependency structure, Figure 9 presents a representative event-specific temporal dependency network.

Operational sensors frequently form densely connected dependency clusters, suggesting recurrent co-variation patterns among equipment-related variables.

Environmental variables occasionally appear as intermediate nodes within the identified dependency structures, indicating their association with both operational and methane-related variables during abnormal evolution processes.

Methane sensors are often located near the terminal portions of the extracted dependency paths, suggesting that methane-threshold exceedance tends to occur together with broader multisensor abnormality patterns observed within the monitored system. Table 8 summarizes the statistical characteristics of the constructed temporal dependency networks.

The constructed temporal dependency networks exhibit moderate sparsity together with relatively stable edge-weight distributions across events. These results suggest that methane exceedance events are associated with recurrent multisensor dependency structures rather than purely random fluctuations.

### 4.5. Evolution Path and Critical Variable Analysis

The identified dependency structures exhibit substantial cross-event consistency rather than purely random fluctuations.

If the inferred temporal dependency networks were dominated by stochastic noise or isolated sensor volatility, the resulting preceding nodes, dependency paths, and critical variables would vary substantially across different methane exceedance events. However, the experimental results reveal recurrent multisensor interaction structures across a large number of independent events.

In particular, several operational variables, including equipment-current-related sensors and working-face operational indicators, repeatedly appear as preceding nodes in dominant dependency paths. Environmental variables such as BA1713 and RH1712 also frequently emerge as intermediate dependency nodes connecting operational disturbances with downstream methane responses.

These recurrent structures suggest that the inferred dependency networks capture stable multisensor abnormal evolution patterns associated with methane exceedance rather than arbitrary statistical fluctuations.

Furthermore, the observed cross-event consistency indicates that methane exceedance evolution is associated with coordinated subsystem interactions involving operational activities, environmental variations, ventilation conditions, and methane-response dynamics.

Therefore, the proposed event-specific dependency analysis framework provides interpretable representations of recurrent abnormal evolution mechanisms in underground monitoring systems rather than isolated event-level visual structures.

To further interpret the structure of methane evolution, both evolution paths and node-level characteristics were analyzed based on the inferred temporal dependency networks.

First, the distribution of the critical node activation count $H_{t}$ was examined to characterize multi-node interaction patterns.

**Figure 10:**
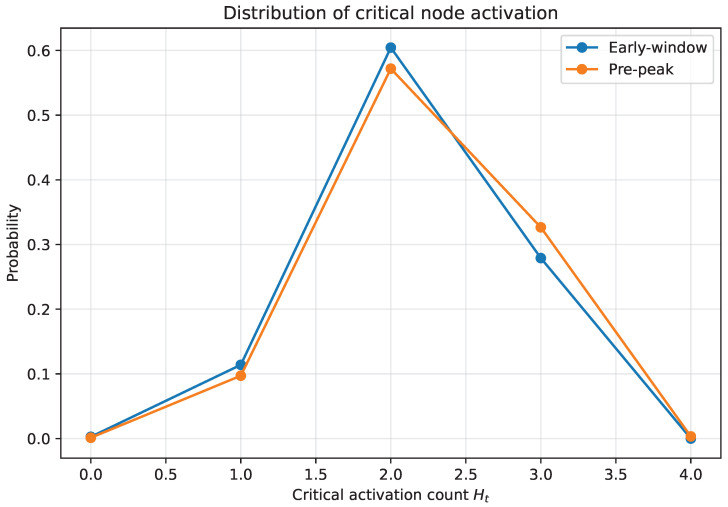
Distribution of critical node activation count $H_{t}$ in early-window and pre-peak phases.

Figure 10 presents the distribution of critical node activation counts $H_{t}$ in the early-window and pre-peak phases. In the early-window phase, the distribution is concentrated at low activation levels, with the probability of $H_{t}=0$ dominating. In contrast, the pre-peak phase exhibits a clear redistribution toward higher activation levels, indicating that coordinated multisensor activation becomes increasingly prominent as the system approaches methane peak events.

These results indicate that methane evolution is associated not only with increasing signal magnitude but also with a transition from isolated behavior to coordinated multi-node activity.

In addition to node activation patterns, evolution paths were analyzed to characterize representative temporal dependency sequences.

**Figure 11:**
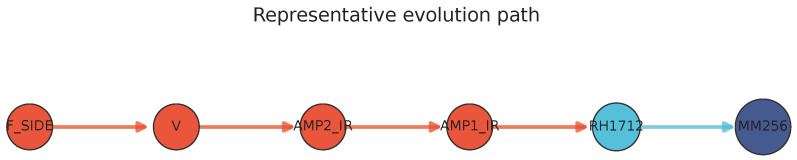
Representative temporal dependency path associated with methane evolution.

Figure 11 illustrates a representative temporal dependency path associated with methane evolution. The identified path starts from operational variables such as F_SIDE, proceeds through equipment-related variables including AMP2_IRand AMP1_IR, and finally reaches the target methane sensor MM256 through environmental variables.

**Figure 12:**
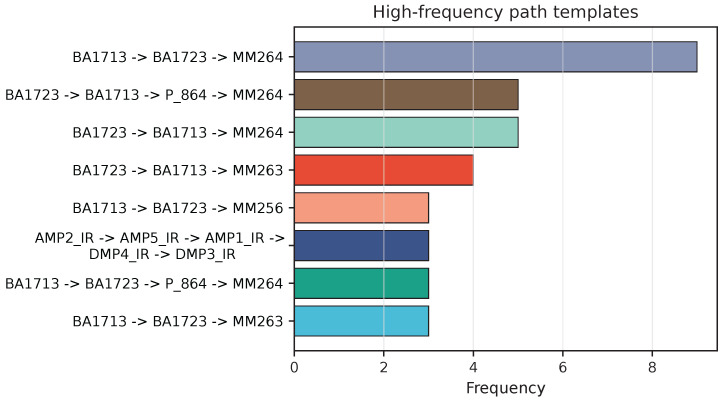
Frequency distribution of dominant evolution paths.

Such sequences provide interpretable descriptions of how multisensor abnormalities are temporally associated within methane exceedance events. These paths should be interpreted as recurrent temporal dependency structures rather than deterministic physical interaction mechanisms or causal relationships. To further examine recurrent structures, the frequency distribution of dominant evolution paths was analyzed across all events, as shown in Figure 12.

Several dependency patterns recur across multiple events. Environmental sensors such as BA1713 and BA1723 frequently appear in temporally preceding positions relative to methane sensors within the extracted dependency paths, suggesting recurrent statistical associations between environmental variations and methane-related abnormalities during event evolution.

To quantify the importance of individual variables, node importance scores were computed based on network centrality and path participation frequency.

**Figure 13:**
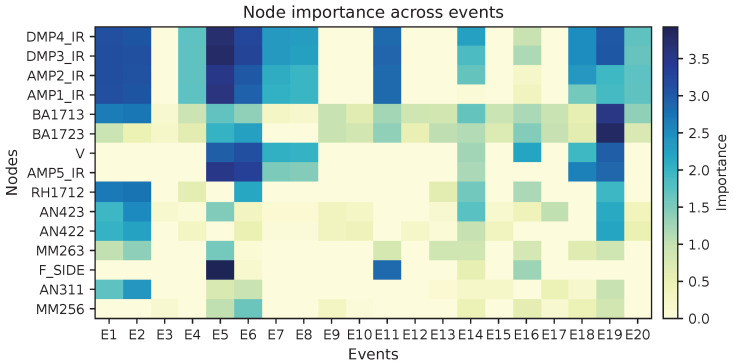
Node importance heatmap across methane exceedance events.

Figure 13 further visualizes node importance distributions across events.

Table 9 lists the top critical variables identified across all events. Several operational and environmental variables consistently occupy central positions across different events, indicating that methane exceedance involves both stable core variables and event-dependent interaction structures.

### 4.6. Sensitivity Analysis

#### 4.6.1. Trigger-Related Parameter Sensitivity

To evaluate the robustness of the proposed early-warning mechanism, sensitivity analyses were conducted under alternative trigger configurations while keeping all remaining components fixed at their baseline values. The trigger mechanism of the proposed framework consists of two functional components: The first component is the risk–threshold pair $\left(\delta_{1},\delta_{2}\right)$, which jointly determines the sensitivity of warning and alarm activation. Since these two thresholds operate together in the trigger process, they were simultaneously increased and decreased by 50% relative to their baseline values to examine the robustness of warning performance.

The second component is the persistence criterion, defined by the persistence window length *L* and the persistence ratio threshold τ. Because these two parameters jointly determine the strictness of persistence requirements, they were also simultaneously perturbed by ±50% from their baseline settings.

For fair comparison, all sensitivity analyses were performed using the same set of event windows as the baseline experiments. Table 10 summarizes the resulting warning performance under alternative trigger configurations, together with the corresponding parameter values.

The results indicate that the proposed framework is relatively insensitive to moderate perturbations of trigger-related parameters. Across all tested settings, the Any-Target effective warning rate varied only between 0.831 and 0.853, while the MM263-specific effective warning rate remained nearly unchanged, ranging from 0.152 to 0.155. The mean effective lead times consistently exceeded 640 s under all configurations.

As expected, decreasing trigger thresholds and relaxing persistence requirements slightly improved event detectability, whereas increasing thresholds and strengthening persistence requirements produced modest reductions in warning performance. Nevertheless, the overall conclusions remained unchanged across all parameter settings. Notably, the baseline configuration did not correspond to the best-performing setting under all evaluation metrics, suggesting that the reported results were not obtained through retrospective parameter optimization. These observations support the robustness of the proposed trigger mechanism under reasonable parameter perturbations.

#### 4.6.2. Dependency Network and Path Sensitivity

To evaluate the robustness of the event-specific temporal dependency analysis, sensitivity analyses were conducted for both dependency-network construction and representative evolution-path identification.

For dependency-network construction, the maximum lag (*MAX_LAG*), edge-retention threshold (θ), and lag-penalty coefficient (β) were individually perturbed by ±50% relative to their baseline values, while keeping all remaining parameters unchanged. Table 11 summarizes the resulting network characteristics and critical-variable rankings under different parameter settings.

The results indicate that the identified critical variables were highly robust to moderate perturbations of the dependency-network parameters. Across all configurations, the Top-10 overlap with the baseline rankings ranged from 0.90 to 1.00, while the Top-5 overlap ranged from 0.80 to 1.00. In particular, the dominant operational variables repeatedly appeared among the highest-ranked nodes under most parameter settings.

As expected, the edge-retention threshold substantially influenced network sparsity, with the mean number of edges ranging from 35.2 to 169.4 across different threshold settings. However, these topological changes had only a limited influence on the overall identification of critical variables. Similarly, perturbations of the maximum lag and lag-penalty coefficient produced only minor variations in node rankings. Overall, these findings suggest that the major interpretative conclusions of the proposed framework are not sensitive to reasonable perturbations of dependency-network parameters.

To further examine whether representative evolution patterns depended on the maximum path-depth constraint, additional analyses were conducted using alternative path-depth settings. In the baseline configuration, the maximum path depth was fixed at $D_{\max}=4$ to balance path interpretability and dependency coverage. Additional experiments were performed using $D_{\max}=3$ and $D_{\max}=5$. The Top-5 dominant paths identified under the three settings were identical, yielding a complete overlap ratio of 1.00. This observation suggests that the representative evolution patterns were not determined by a specific choice of the maximum path-depth constraint.

This result is also consistent with the product-based path-scoring formulation adopted in this study. Because longer paths accumulate multiplicative penalties through successive edge weights, extending the maximum allowable path depth does not necessarily promote longer dependency chains to the highest-ranking positions. Therefore, the baseline choice of $D_{\max}=4$ provides a reasonable balance between interpretability and dependency coverage without materially affecting the representative evolution patterns.

## 5. Conclusions

This study proposes an interpretable event-driven multisensor risk-evolution analysis framework for methane early warning in underground coal mine monitoring systems. Instead of treating methane exceedance as an isolated prediction target, the proposed framework models methane warning events as progressive multisensor abnormal evolution processes involving operational, ventilation, environmental, methane-drainage, and methane-response subsystems.

A continuous multisensor risk representation together with a persistence-based trigger mechanism was first developed to identify sustained abnormal evolution prior to methane threshold exceedance. Event-specific temporal dependency networks were subsequently constructed to characterize lagged multisensor interaction structures within methane exceedance windows. Based on the inferred dependency structures, representative evolution paths and recurrent critical variables were further identified across events to provide interpretable descriptions of abnormal evolution mechanisms.

Experiments conducted on a real underground coal mine monitoring dataset containing 784 methane exceedance events demonstrated that the proposed framework achieved improved effective early-warning capability compared with multiple statistical process-monitoring and anomaly-detection baselines. The results further showed that methane exceedance events are generally associated with structured multisensor abnormal evolution processes rather than isolated methane fluctuations. Operational and environmental variables repeatedly emerged as dominant upstream components in temporal dependency networks, indicating the existence of recurrent multi-subsystem interaction patterns during methane evolution.

Compared with conventional prediction-oriented approaches, the proposed framework emphasizes interpretable system-level abnormal evolution analysis rather than purely predictive accuracy. The framework therefore provides a complementary perspective for industrial early warning by enabling analysis of trigger formation, temporal dependency development, and recurrent precursor structures associated with methane exceedance events.

Sensitivity analyses further demonstrated that the major conclusions remained stable under moderate perturbations of trigger-related parameters, dependency-network construction parameters, and path-depth settings. Although network sparsity and warning sensitivity varied as expected under different configurations, the identified critical variables, representative evolution structures, and overall early-warning conclusions remained largely unchanged. These findings support the robustness of the proposed framework and suggest that the reported results were not dependent on retrospective parameter optimization.

Several limitations remain in the current study. First, the constructed dependency networks represent statistically inferred temporal associations rather than strict physical causality or methane transport mechanisms. Second, several framework parameters were empirically selected according to underground monitoring characteristics. Third, the experiments were conducted using data from a single monitoring environment, and cross-site generalization requires further investigation. In addition, the present study adopted an event-centered retrospective analysis strategy to facilitate interpretable investigation of methane exceedance evolution processes. Although this design enables detailed characterization of precursor evolution patterns, further validation under continuous online monitoring scenarios with explicit false-alarm evaluation is still required.

Future work will focus on integrating adaptive dependency learning, causal interaction analysis, and cross-mine transfer validation to further improve the robustness and generalizability of interpretable multisensor early-warning analysis in complex industrial systems.

## Figures and Tables

**Table 1:** Sensor-level risk-direction assignments used for anomaly aggregation.

Category	Sensors	$s_{i}$	Engineering Rationale
Methane	MM252, MM261, MM262, MM263, MM264, MM256, MM211, CM861	+1	Higher methane concentration directly indicates elevated methane accumulation risk.
Ventilation	AN311, AN422, AN423	−1	Reduced airflow may weaken methane dilution capacity and increase methane accumulation risk.
Methane drainage	CR863, P_864, TC862, WM868	−1	Reduced drainage effectiveness may decrease gas extraction efficiency and increase methane accumulation risk.
Environmental	TP1711, RH1712, BA1713, TP1721, RH1722, BA1723	+1	Abnormal environmental deviations may accompany changes in underground operating conditions associated with methane-evolution processes.
Operational	AMP1_IR, AMP2_IR, DMP3_IR, DMP4_IR, AMP5_IR, V	+1	Abnormal operational variations may coincide with disturbances associated with abnormal system evolution.
Operational (Feeder)	F_SIDE	+1	Abnormal feeder-state variations may coincide with operational disturbances associated with abnormal system evolution.

**Table 2:** Sensors included in the monitoring dataset.

Category	Sensors	Description
Ventilation	AN311, AN422, AN423	Airflow velocity in mine tunnels
Environment	TP1721, RH1722, BA1723, TP1711, RH1712, BA1713	Temperature, humidity, and pressure
Methane	MM252, MM261, MM262, MM263, MM264, MM256, MM211, CM861	Methane concentration sensors
Methane drainage	CR863, P_864, TC862, WM868	Drainage pressure and flow conditions
Operation	AMP1_IR, AMP2_IR, DMP3_IR, DMP4_IR, AMP5_IR, F_SIDE, V	Equipment operating conditions

**Table 3:** Statistical characteristics of methane exceedance events.

Event Type	Threshold (%CH_4_)	Number of Events	Average Duration (s)	Median (s)	Min	Max
Warning	1.000	705	551.2	174.0	2	18,569
Alarm	1.500	79	210.5	68.0	2	2632

**Table 4:** Benchmark comparison of early-warning performance.

Method	Any-Target Raw Rate	Any-Target Effective Rate	MM263 Raw Rate	MM263 Effective Rate	Mean Effective Lead Time (s)	Median Effective Lead Time (s)
Proposed-Full-Evolution	0.925	0.848	0.165	0.154	660.8	600
One-Class-SVM	0.806	0.775	0.147	0.144	586.1	511
Shewhart-Z	0.856	0.791	0.152	0.139	593.6	568
PCA-SPE	0.808	0.760	0.144	0.139	553.4	500
KMeans-Distance	0.805	0.758	0.147	0.139	543.2	499
CUSUM	0.793	0.760	0.144	0.137	571.4	510
PCA-T_2_	0.746	0.691	0.137	0.132	528.3	494
Local-Outlier-Factor	0.686	0.638	0.127	0.120	547.4	493
Isolation-Forest	0.544	0.509	0.114	0.106	527.7	473
Rate-of-Change	0.477	0.412	0.060	0.042	694.7	600

**Table 5:** Summary of Any-Target-Sensor and sensor-specific warning results.

Target Sensor	Warning Windows	Raw Warnings	Effective Warnings	Effective Rate Within Warning Windows
Any-Target	599	554	508	0.848
MM263	99	96	92	0.929
MM264	297	282	258	0.869
MM256	338	311	292	0.864

**Table 6:** Occupancy characteristics of warning and alarm states.

Metric	Value
Warning Occupancy Ratio	2.23%
Alarm Occupancy Ratio	0.13%
Combined Occupancy Ratio	2.36%
Mean Warning Duration (s)	31.8
Mean Alarm Duration (s)	1.9

**Table 7:** Event-level McNemar test results under the Any-Target-Sensor evaluation criterion.

Comparison	*p*-Value
Proposed vs. Shewhart-Z	$1.14\times 10^{-13}$
Proposed vs. One-Class-SVM	$1.11\times 10^{-16}$
Proposed vs. PCA-SPE	$2.17\times 10^{-19}$
Proposed vs. KMeans-Distance	$1.08\times 10^{-19}$
Proposed vs. CUSUM	$2.17\times 10^{-19}$
Proposed vs. PCA-T^2^	$9.86\times 10^{-32}$
Proposed vs. Local-Outlier-Factor	$2.30\times 10^{-41}$
Proposed vs. Isolation-Forest	$1.52\times 10^{-64}$
Proposed vs. Rate-of-Change	$5.27\times 10^{-82}$

**Table 8:** Statistical characteristics of temporal dependency networks.

Metric	Value	Description
Number of Event Networks	784.0	Events containing at least one dependency edge
Unique Nodes	28.00	Distinct sensors appearing in event networks
Average Edge Count	72.22	Average number of edges per event network
Median Edge Count	64.00	Median number of edges per event network
Network Density	0.096	Ratio of observed to possible directed edges
Average Edge Weight	0.339	Mean dependency strength across edges
Median Edge Weight	0.276	Median dependency strength across edges

**Table 9:** Top critical variables in temporal dependency networks.

Node	Mean Imp.	Median Imp.	Max Imp.	Out-Str.	In-Str.	Betweenness	Freq.
DMP3_IR	2.180	2.149	5.147	3.272	2.887	0.016	0.607
DMP4_IR	2.145	2.091	5.197	3.205	2.864	0.014	0.610
AMP5_IR	2.116	2.164	5.820	3.196	2.771	0.022	0.459
AMP2_IR	1.943	1.885	5.847	2.875	2.627	0.016	0.599
AMP1_IR	1.927	1.828	5.087	2.895	2.550	0.013	0.598
V	1.895	1.822	5.269	2.842	2.485	0.043	0.420
F_SIDE	1.542	1.370	5.778	2.344	1.974	0.041	0.302
RH1712	1.337	1.192	5.053	2.156	1.523	0.059	0.504
BA1713	1.254	1.109	5.158	1.955	1.511	0.061	0.962
P_864	1.208	1.067	5.035	1.672	1.737	0.062	0.724

**Table 10:** Sensitivity analysis under alternative trigger configurations.

Trigger Configuration	Any-Target Raw Rate	Any-Target Effective Rate	MM263 Raw Rate	MM263 Effective Rate	Mean Effective Lead Time (s)	Median Effective Lead Time (s)
Baseline Setting L=12,τ=0.50 $\delta_{1}=0.30,\;\delta_{2}=0.55$	0.925	0.848	0.165	0.154	660.8	600
Relaxed Persistence L=6,τ=0.40	0.930	0.853	0.169	0.152	675.8	612
Strict Persistence L=24,τ=0.70	0.902	0.838	0.159	0.152	644.2	576
Low Threshold Pair $\delta_{1}=0.20,\;\delta_{2}=0.50$	0.927	0.850	0.165	0.155	668.5	604
High Threshold Pair $\delta_{1}=0.40,\;\delta_{2}=0.70$	0.907	0.831	0.160	0.154	642.8	589

**Table 11:** Sensitivity analysis under alternative dependency-network configurations.

Network Configuration	Mean Edges	Median Edges	Top-5 Overlap	Top-10 Overlap
Baseline Setting $\ell_{\max}=5$ θ=0.20,β=0.20	72.2	64	1.00	1.00
Reduced Lag Range $\ell_{\max}=3$	65.1	57	1.00	1.00
Extended Lag Range $\ell_{\max}=8$	79.2	72	1.00	0.90
Low Edge Threshold θ=0.10	169.4	156	0.80	0.90
High Edge Threshold θ=0.30	35.2	29	1.00	0.90
Low Lag Penalty β=0.10	72.2	64	0.80	1.00
High Lag Penalty β=0.30	72.2	64	1.00	1.00

## Data Availability

The dataset analyzed in this study is publicly available from the Mendeley Data repository: https://data.mendeley.com/datasets/yd7vw4c5mk/1 (accessed on 3 March 2026). The data are openly accessible for academic research and reproducibility purposes.
